# Effects of gamma-aminobutyric acid and piperine on gene regulation in pig kidney epithelial cell lines

**DOI:** 10.5713/ajas.19.0745

**Published:** 2019-12-24

**Authors:** Juhyun Shin, Yoon-Mi Lee, Jeongheon Oh, Seunghwa Jung, Jae-Wook Oh

**Affiliations:** 1 Department of Stem Cell and Regenerative Biotechnology, KIT, Konkuk University, Seoul, 05029 Korea

**Keywords:** Gamma-aminobutyric Acid, Piperine, Pig Kidney Epithelial Cell, Microarray, Erythrocyte Differentiation, Immune System, MAPK Pathway

## Abstract

**Objective:**

Gamma-aminobutyric acid (GABA) and piperine (PIP) are both nutritional supplements with potential use in animal diets. The purpose of this study is to investigate the effect of GABA and/or PIP treatment on the gene expression pattern of a pig kidney epithelial cell line.

**Methods:**

LLCPK1 cells were treated with GABA, PIP, or both, and then the gene expression pattern was analyzed using microarray. Gene ontology analysis was done using GeneOntology (Geneontology.org), and validation was performed using quantitative real-time polymerase chain reaction.

**Results:**

Gene ontology enrichment analysis was used to identify key pathway(s) of genes whose expression levels were regulated by these treatments. Microarray results showed that GABA had a positive effect on the transcription of genes related to regulation of erythrocyte differentiation and that GABA and PIP in combination had a synergistic effect on genes related to immune systems and processes. Furthermore, we found that effects of GABA and/or PIP on these selected genes were controlled by JNK/p38 MAPK pathway.

**Conclusion:**

These results can improve our understanding of mechanisms involved in the effect of GABA and/or PIP treatment on pig kidney epithelial cells. They can also help us evaluate their potential as a clinical diagnosis and treatment.

## INTRODUCTION

Gamma-aminobutyric acid (GABA) is one of inhibitory neurotransmitters known to be synthesized in the brain from glutamic acid via glutamic acid decarboxylase [1]. Its dysregulation can potentially result in seizure. Therefore, it is a common target to treat epilepsy [2]. GABA is known to act through its ionotropic type-A and -C receptors (GABA_A_ and GABA_C_ receptors) [3] and its metabotropic type-B receptor (GABA_B_ receptor) [4]. GABAergic system, which involve the interaction of the neurotransmitter GABA and its receptors, was first discovered in the neuron of the brain tissue [5], but was shown to be present in several organs including kidney [6].

Piperine (PIP) is a major alkaloid present in seeds of black pepper ( *Piper nigrum*), long pepper (*P. longum*), and other pepper species (Piperaceae). It is responsible for pepper seeds’ characteristic biting taste [7]. Beside its dietary function, PIP is long known to have various physiological benefits including anti-inflammatory, anti-cancer, anti-oxidant, and bioavailability-enhancing activities [8]. Moreover, PIP has been proposed as an alternative to antibiotic growth promoter in animal feedings [9]. PIP can modulate GABA_A_ receptors [10]. We have recently demonstrated that PIP and GABA have synergistic effect in pig kidney cell line LLC-PK1, resulting in increased erythropoietin (*EPO*) and *EPO* receptor (R) expression possibly via the activation of MAPK signaling cascade through GABA_A_ receptor mediated signaling [11].

*EPO* is a pleiotropic cytokine that can affect infection and inflammation in kidney [12]. Thus, we hypothesized that GABA and PIP might be useful for treating or preventing possible kidney related diseases and infection. However, beside *EPO* and *EPO-R* upregulation, little is known about differential expression profiles of the genes following GABA and PIP treatment in kidney epithelial cells.

Thus, the objective of this research was to perform micro array analysis to determine effects of GABA and PIP on gene expression of pig kidney epithelial cells. Expression levels of genes in key pathways were validated. Results revealed that genes affected by GABA and PIP in expression level were associated with MAPK pathway. These results provide new insight into effects of GABA and PIP. They could be used to evaluate their impact on kidney epithelial cells.

## MATERIALS AND METHODS

### Cell lines culture and treatment

LLC-PK1 (ATCC CL101) was obtained from the American Type Culture Collection (Manassas, VA, USA). Cells were cultured at 37°C with 5% CO_2_ in Dulbecco’s modified Eagle’s medium (Hyclone, Logan, UT, USA) with 8% Fetal Bovine Serum, 100 U/mL penicillin, and 100 μg/mL streptomycin (Welgene, Daegu, Korea). Treatments with GABA and PIP (Sigma Aldrich, St. Louis, MO, USA), MAPK inhibitors SB 202190 and SP600125 (Calbiochem, San Diego, CA, USA) was performed when cells reached 80% to 90% confluence. Cells were incubated at 37°C with 5% CO_2_ in DMEM without fetal bovine serum for 1 hour before treatments with 0.1 mM GABA and/or 5 μM PIP in final concentration.

### RNA isolation

Prepared cells were harvested and RNAs were isolated using TRIzol reagent (Ambion, Carlsbad, CA, USA) or RNeasy min kit (Qiagen, Germantown, MD, USA). The purity and integrity of total RNA were checked using NanoDrop One (Thermo Scientific, Waltham, MA, USA).

### Microarray analysis

cRNA was prepared from 1 to 5 μg total RNA using Agilent’s Quick Amp labeling kit (Agilent, Santa Clara, CA, USA). Labeling of probes, *in vitro* transcription, hybridization, and dyeing of chips were performed by Macrogen Gene Company Limited (Seoul, Korea). Then 1.65 μg of cRNA was hybridized to Agilent Porcine Gene Expression microarray (43,803 probes). Arrays were scanned for data export and processing using Agilent Feature Extraction v11.0.1.1 software on R 2.15.1. Shortly after, signal intensity was extracted. Quality check and filtering were performed by flag. Probe signal intensity was converted to log (base 2) value. Normalization for untreated, GABA, PIP, or both was performed using quantile normalization. Fold changes (FC) compared to untreated sample were calculated. Probes that yielded >1.5 or <−1.5 folds change with a p-value <0.05 in either treatment relative to untreated LLC-PK1 were retained, totaling 2,393 probes.

### Differential gene expression analysis

Heatmapper [13] was used to generate heatmap plot representing genes using average linkage clustering method with Pearson measurement method. To evaluate biological functions of differentially expressed gene, gene ontology analysis was performed using statistical analysis tool PANTHER Overrepresentation Test [14] (Released 20190308) with GeneOntology database released 2019-01-01 and *Sus scrofa* (all genes) as reference. Fischer’s exact test with Bonferroni correction for multiple testing (p<0.05) was performed with the set of validated genes from microarray data. Functional groups were ordered by their overall gene numbers and overall enrichment score [15].

### Quantitative real-time reverse-transcription polymerase chain reaction and statistical analysis

To validate selected differentially expressed genes, real-time reverse-transcription polymerase chain reaction (RT-PCR) was performed using CFX96 Real-Time System (BIO-RAD, Hercules, CA, USA). Briefly, 1 μg of RNA was treated with DNA-free Kit (Thermo Scientific, USA) following the manufacturer’s protocol. Subsequently, first-strand DNA synthesis was conducted with oligo [13] and AccuPower CycleScript RT PreMix kit (BIONEER, Daejeon, Korea). Amplification was done using IQ SYBR Green Supermix (BIO-RAD, USA). Primers used are listed in Table 1. The results were reported as FC compared to control expression normalized to glyceraldehyde 3-phosphate dehydrogenase by the Pfaffl method [16]. Pairwise comparisons between means of different treatments were performed using a two tailed Student *t*-test, the null hypothesis being that the means are equal.

## RESULTS

### Differentially expressed genes in kidney cell after treatment with GABA, PIP, or both

**Figure 1:**
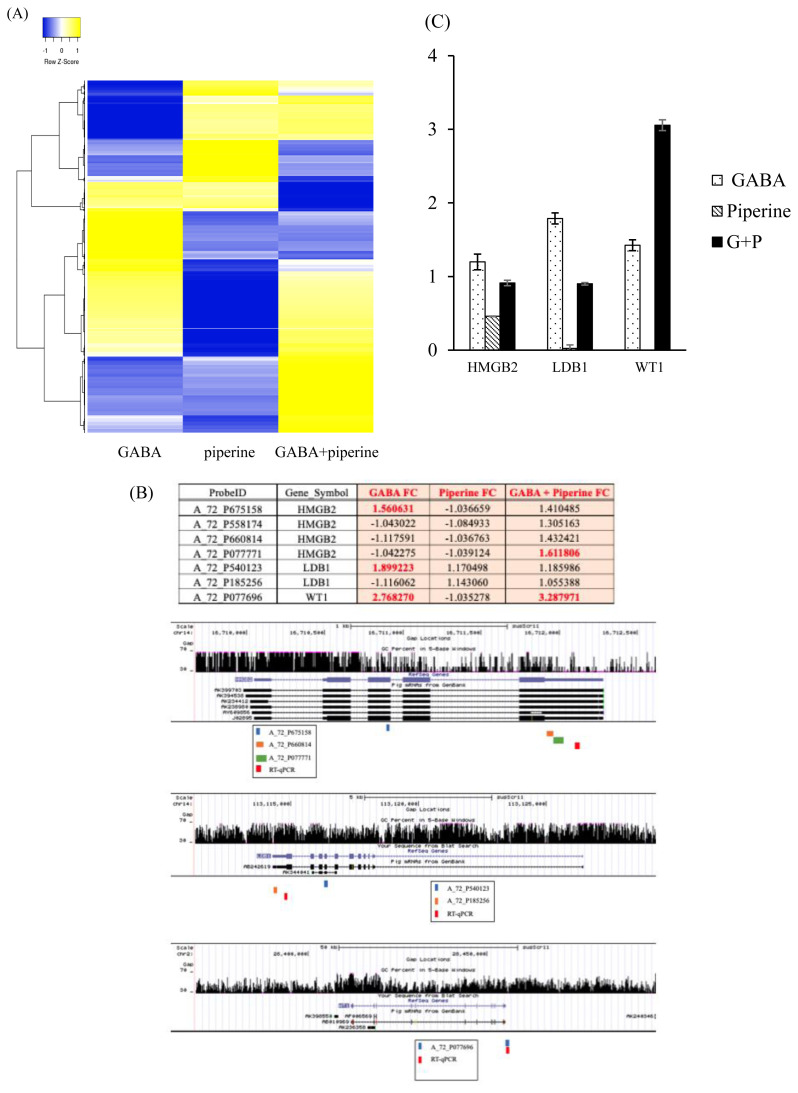
Hierarchical clustering of dataset of expressional changes upon treatment with GABA, PIP, or both in LLCPK1 kidney cells based on 2,393 probes. Heatmap rows were clustered by average linkage and Euclidean cluster (A). Table of probes in Agilent Porcine Gene Expression microarray represent genes of interest with FC, location of probes, and primer designed in genes according to UCSC gene browser (B). RT-qPCR validation of genes of interest upon treatment with GABA, PIP, or both (C). GABA, gamma-aminobutyric acid; PIP, piperine; FC, fold changes; UCSC, University of California Santa Cruz; RT-qPCR, quantitative reverse transcription polymerase chain reaction.

Differential expressed genes heatmap has been plotted in Figure 1A. For validation purpose, FCs of genes of interest were retrieved as differential signals from multiple probes might reflect differential isotype expression upon treatment [17]. Probes were located and mRNA isotypes of the genes of interest were analyzed using UCSC genome Browser (http://genome.ucsc.edu/) based on *Sus Scrofa* genome (Sscrofa11. 1/susScr11) (Figure 1B). High mobility group protein B2 (HMGB2) was represented by five probes. However, validation showed that only four probes corresponded to *HMGB2*. Three of these probes were located in the 3′ UTR and one probe was located in the third exon of identified *HMGB2* isotypes. Of these four probes, A_72_P675158 was upregulated by GABA and A_72_P077771 was synergistically upregulated by GABA and PIP at significant levels while the remaining two showed synergistic upregulation at lower magnitude (FC<1.5). LIM domain-binding protein 1 (*LDB1*) was represented by two probes. A_72_P540123 located in the seventh exon of AB242619 or the second exon of AK344041 showed upregulation upon GABA treatment. On the other hand, A_72_P185256 located in the 5′ UTR of AB242619 showed no significant change upon GABA and/or PIP treatment. Only one probe for Wilms tumor protein homolog (*WT1*) was available. A_72_P077696 located in the 3′ UTR of the longest isotype showed upregulation by GABA alone and in combination with PIP. To validate these results, we designed quantitative RT-PCR (RT-qPCR) primers in the 3′UTR of reference sequences of *HMGB2*, *LDB1*, and *WT1* according to NCBI RNA reference sequences collection (RefSeq). After GABA and/or PIP treatment at the same condition as for microarray analysis, mRNAs were extracted and RT-qPCR was performed to evaluate expression levels of *HMGB2*, *LDB1*, and *WT1* after GABA and/or PIP treatment compared to untreated cells (Figure 1C). *HMGB2* and *LDB1* showed increase by GABA treatment as A_72_P675158 and A_72_P540123 probes. WT1 showed expression pattern that concurred with its microarray probe.

### Gene ontology analysis of genes regulated by GABA and/or PIP treatment

**Figure 2:**
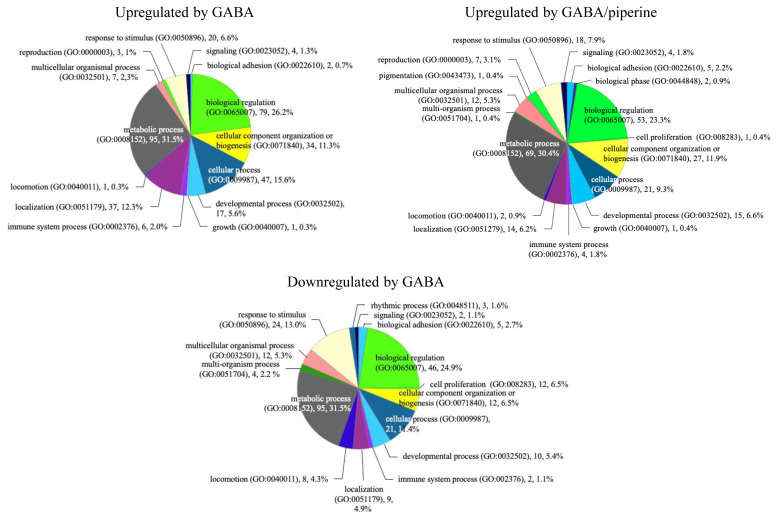
Functional annotation chart showing distribution of genes regulated by GABA or GABA/PIP co-treatment in LLC-PK1 cells based on PANTHER. GABA, gamma-aminobutyric acid; PIP, piperine.

From microarray data, we identified 302 genes upregulated by GABA alone and 26 genes upregulated by PIP alone (FC >1.5). There were 202 downregulated genes by GABA alone and 43 downregulated genes by PIP alone. Synergistically upregulated genes were defined by two criteria. First, we filtered for genes with FCs greater than 1.5 upon GABA and PIP co-treatment. Second, among these genes, we selected 227 genes that has higher FC after co-treatment compared to treatment by GABA alone or PIP alone. Twenty genes were defined as synergistically downregulated as FCs after co-treatment were <−1.5 while FCs after GABA or PIP treatment were between 1.0 and 1.1. GO analysis was performed for genes upregulated by GABA, GABA/PIP, and downregulated by GABA alone. We were unable to perform GO analysis for lists of genes affected by PIP treatment and genes downregulated by GABA/PIP in combination due to low number of genes in these lists (Figure 2). GABA-upregulated genes were related to metabolic process (31.5%), biological regulation (26.2%), cellular process (15.6%), localization (12.3%), and cellular component organization or biogenesis (11.3%). GABA- and PIP- upregulated genes were related to metabolic process (30.4%), biological regulation (23.2%), cellular component organization or biogenesis (11.9%), and cellular process (9.3%). Genes downregulated by GABA alone were related to metabolic process (31.5%), biological regulation (24.9%), response to stimulus (13.0%), and cellular process (11.4%).

### Fold enrichment analysis of differentially expressed genes

**Figure 3:**
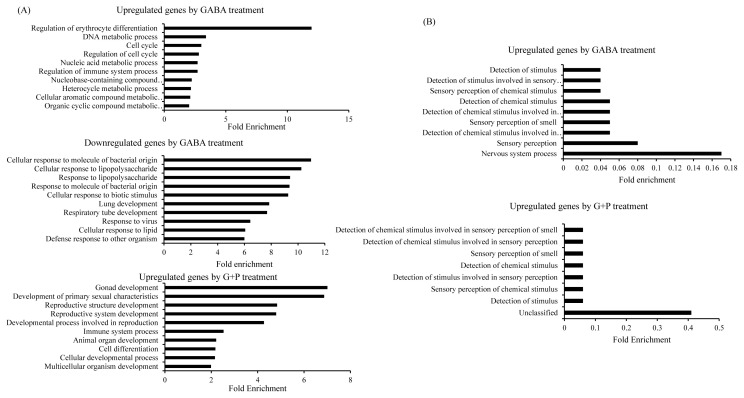
Fold enrichment plot showing biological processes positively enriched in GABA- upregulated, GA A-downregulated, and GABA/PIP co-treatment synergistically upregulated gene lists (A). Fold enrichment plot showing underrepresented biological processes in GABA and GABA/PIP co-treatment upregulated gene lists (B). Biological terms were identified by genes with respect to biological process through AmiGO. GABA, gamma-aminobutyric acid; PIP, piperine.

Fold enrichment analysis can evaluate the number of genes that represent a specific pathway versus the number expected at random case [14]. At a p-value cut-off of 0.05, 15 biological processes were enriched at >1.89- fold for GABA upregulated genes, 76 biological processes were enriched at >2.00-fold for GABA downregulated genes, and 15 genes were enriched at >1.80-fold for synergistically upregulated genes (Figure 3A). The most upregulated biological pathway by GABA alone treatment was erythrocyte differentiation, while the majority of downregulated biological pathways were involved in responses to various stimulus. The most upregulated biological pathway in synergistically upregulated was related to reproductive development. In contrast to fold enrichment higher than one, fold enrichments that were lower than one represented biological processes that were underrepresented and therefore putatively downregulated in the gene list of interest (Figure 3B). At p-value cut-off of 0.05, nine biological processes were enriched at <0.18-fold for GABA upregulated genes and seven biological pathways were enriched at <0.10-fold for GABA/PIP upregulated gene. Both lists showed underrepresentation of biological processes related to stimulus.

### Validation and analysis of genes related to biological pathways upon treatment with GABA or GABA/PIP in combination

**Figure 4:**
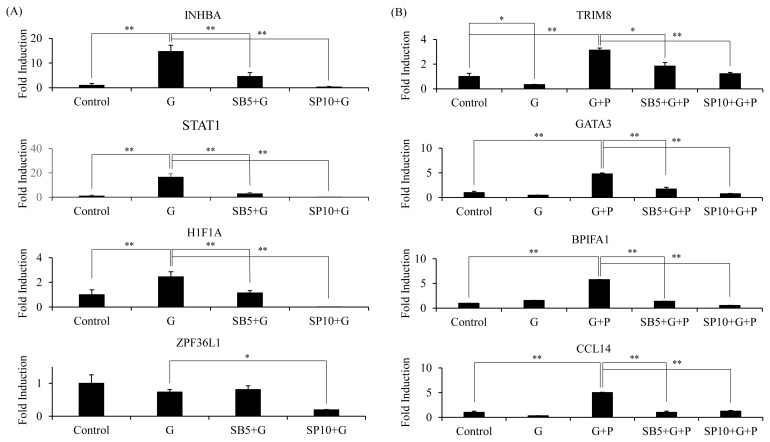
RT-qPCR analysis of genes upregulated by GABA alone that are involved in erythrocyte differentiation (GO:0045648) (A) and genes synergistically upregulated by GABA and PIP co-treatment that are involved in regulation of immune system process (GO:0002682) (B). Cells [39] were treated with GABA (G), PIP (P), or both (G+P) as described previously. 5 μM MAPK inhibitor SB203580 (SB5) and 10 μM of MAPK inhibitor SP600125 (SP10) were added at 1 hour prior to treatment with GABA, PIP, or both. Relative expression to control was normalized to GAPDH expression. RT-qPCR were performed three times and representative experiments were presented. RT-qPCR, quantitative reverse transcription polymerase chain reaction; GABA, gamma-aminobutyric acid; PIP, piperine; GAPDH, glyceraldehyde-3-phosphate dehydrogenase. Two tailed student t-test was performed, * p<0.01, ** p<0.001.

Based on microarray data and enrichment analysis, we identified seven genes that were upregulated by GABA and related to biological process controlling positive regulation of erythrocyte differentiation. We also identified 31 genes that were synergistically upregulated and related to immune systems and processes. FCs of these genes of interests were retrieved from microarray data (Table 2). Validation was performed for inhibin subunit beta A (*INHBA*), signal transducer and activator of transcription 1 (*STAT1*), hypoxia-inducible factor 1-alpha (*HIF-1alpha*), and SPF36 ring finger protein like 1 (*ZPF36L1*) among GABA upregulated genes. Our previous study has shown that *EPO* and *EPO-R* upregulation by GABA/PIP in combination can be downregulated by inhibitor of JNK (SP600126) and inhibitor of p38 (SB203580) MAPK [11]. Therefore, we investigated if MAPK inhibition could affect GABA upregulated genes (Figure 4A). *INHBA*, *STAT1*, and *HIF1A* were validated to be upregulated by GABA. Inhibition of JNK activation significantly downregulated expression of these genes. However, inhibition of p38 activation affected expression of these genes at lesser extent. Validation was performed for tripartite motif containing 8 (*TRIM8*), GATA binding protein 3 (*GATA3*), BPI fold containing family A member 1 (*BPIFA1*), and C-C motif chemokine ligand 4 (*CCL4*) among synergistically upregulated genes involved in biological process related to immune system process. Expression analysis upon addition of inhibitors was also performed for those genes (Figure 4B). Results showed that *TRIM8*, *GATA3*, *BPIFA1*, and *CCL4* were synergistically upregulated by GABA and PIP in combination, while the addition of MAPK inhibitors downregulated their expression.

## DISCUSSION

Neurotransmitter GABA has been widely studied in human patients as treatment for neuronal diseases including Alzheimer’s disease [18]. In addition, several studies have shown that GABA plays an important role in non-neuronal tissues and that it can be used to treat diseases including liver injury [19]. In kidney, GABA is shown to have anti-inflammatory properties and promote fibroblast proliferation [20]. It can be used to treat renal dysfunction by controlling blood pressure and lipid profile [21], demonstrating its pharmacological potential. In dairy cows, dietary supplementation of GABA has been shown to improve the food intake, lactation performance, and health of early lactating calves [22]. In Hanwoo steers, GABA supplementation improved *in vitro* rumen fermentation and lowered blood endotoxin levels [23]. Similarly, GABA improved growth performance of weaned pigs [24].

It has been previously shown that plant derived alkaloid PIP can affect GABA_A_ receptor, thus affecting GABA induced signals [11]. Moreover, PIP has been proposed as an alternative growth promoter to antibiotics in animal diets [9,25]. However, while its beneficiary effects in poultry has been investigated [25–28], its impact on porcine health remains relatively elusive. In previous study, we have previously demonstrated that expression of *EPO* and *EPO-R* is upregulated by GABA and PIP in combination in epithelial kidney cell lines through MAPK pathway [11].

In this study, we analyzed microarray data of differential gene expression upon treatment with GABA and/or PIP to improve our understanding of affected processes. We observed that the main process affected by treatment with GABA or GABA/PIP was biological regulation. Interestingly, the percentage of genes related to response to stimulus among genes downregulated by GABA was higher than other treatment. Moreover, fold enrichment analysis showed that response to stimulus was one of the most underrepresented pathways in genes upregulated by GABA. This suggests that GABA affects stimulus response in kidney. Therefore, the anti-stress effect after GABA treatment might not be limited to neural cells.

Fold enrichment analysis revealed a group of genes that represented putative key pathway affected by GABA and/or PIP treatments. GABA treatment greatly affected seven genes related to erythrocyte differentiation. Of these seven genes, three were validated in our study. *INHBA*, also known as erythroid differentiation protein, was shown to be involved in several pathways such as neoplastic activity beside erythroid differentiation [29]. *STAT1* has been shown to be able to rescue *GATA1* knock out phenotype (failure of differentiation of hematopoietic progenitors into erythrocytes and megakaryocytes) in mice [30]. *HIF1A* is a transcription factor activated in response to hypoxia. It can induce *EPO* expression [31]. Our findings showed that GABA could induce expression of those genes in kidney cells, suggesting that GABA treatment might be effective as a treatment or supplement to treat diseases, such as well-known anemia occurring in growing piglet [32].

Surprisingly, only a few genes were significantly upregulated or downregulated (26 and 43, respectively) by PIP treatment alone. No specific biological process was enriched at significant level by PIP treatment alone. This suggests that PIP might have less effect on kidney cells than GABA. Another possibility was that we treated cells with a relatively low PIP concentration in this study. Effects of PIP treatment might not have been assayed at its full potential. Indeed, to investigate GABA/PIP synergistic effect, when we treated LLC-PK1 cells with 5 μM PIP. It has been reported that PIP has no toxicity at concentration up to 50 μM [11]. Therefore, to investigate the full potential effect of PIP treatment on the kidney, further analysis with higher concentration might be needed.

As our previous results indicated that GABA and PIP in combination increased expression levels of *EPO* and *EPOR*, we investigated biological processes of additional genes upregulated by GABA and PIP in combination. According to fold enrichment analysis, GABA and PIP in combination increased expression level of genes related to reproductive tissue development. Although co-treatment might have major role in reproductive development, some genes that were designated to gonad development (GO:0008406) and reproductive structure development (GO: GO:0048608) such as *GATA3* and *WT1* were also related to erythrocyte or *EPO* expression [33]. We have previously shown that GABA and PIP in combination have a synergistic effect in increasing *EPO* and *EPO-R* expression and that kidney is one of the main organs responsible for EPO synthesis [34]. Thus, GABA and PIP in combination might have a synergistic role in increasing red blood cell production. Beside process related to reproductive development, GABA and PIP in combination synergistically upregulated genes related to immune system process. *TRIM8* is a TRIM protein known to positively regulate nuclear factor-κB activation [35]. *GATA3* is a key transcription factor in immune regulation [36]. *BPIFA1* is a secreted protein that has a protective role during infection [37]. *CCL4* is a chemokine related to pro-inflammatory response [38]. We validated these genes’ upregulation in the present study. Moreover, we showed that these synergistically upregulated genes were controlled by MAPK pathway, like *EPO*, *EPO-R*, and GABA upregulated genes.

In conclusion, we identified genes upregulated by GABA, PIP, or both in kidney cells. GABA affected several biological pathways, including the expected erythrocyte differentiation pathway. GABA lowered expression levels of genes related to stimulus, showing that GABA treatment might have anti-stress effect on kidney cells. While PIP treatment was previously shown to affect GABA_A_ receptor, its effect was not as extensive as GABA. However, co-treatment increased expression levels of genes related to reproductive systems, blood cell developments, and immune system. This suggests that GABA and PIP in combination might increase response of aforementioned processes through MAPK pathway.

## Figures and Tables

**Table 1:** List of primers used in this study

Primer	Primer sequence (5′ to 3′)
Pig_BPIFA1_96F	cccccactgggatgactgctccaga
Pig_BPIFA1_96R	gggagtgctggcaccaccgctgta
Pig_CCL14_88F	ccagcagccagtgccccaagcct
Pig_CCL14_88R	acccagtcatccctggggttggcac
Pig_GAPDH_qPCR_F_144	gcgagaactgcctttctgag
Pig_GAPDH_qPCR_R_144	aaggttgcctcgtttgtctg
Pig_GATA3_qPCF_106F	tcggcagcgcgaagggcaggt
Pig_GATA3_qPCF_106R	gcccacaggcattgcagacggggt
Pig_HIF1A_qPCR_F_99	atggaacggagcaaaagaca
Pig_HIF1A_qPCR_R_99	tggtcagctgtggtaatcca
Pig_HMGB2_qPCR_F_123	agtgcaggttgcagcttttt
Pig_HMGB2_qPCR_R_123	cgagtttgctgttaccatacaca
Pig_INHBA_qPCR_F_104	aaaggtgtgggacagaggtg
Pig_INHBA_qPCR_R_104	ttgcaatacacgggactgaa
Pig_LDB1_qPCR_F_99	ggcctctgagaaatgtcctg
Pig_LDB1_qPCR_R_99	cttgaaggggatggagtcag
Pig_STAT1_qPCR_F_104	ttcttcctgaacccaccttg
Pig_STAT1_qPCR_R_104	ttcagctggtccacattgag
Pig_TRIM8_80F	tggccaagaaggagaagcagctgcgga
Pig_TRIM8_80R	ggggacgctctgcaggaagggca
Pig_WT1_qPCR_F_106	cgctctcaaagaaggaaacg
Pig_WT1_qPCR_F_106	cgctctcaaagaaggaaacg
Pig_WT1_qPCR_R_106	agcagaggaccaactcctca
Pig_WT1_qPCR_R_106	agcagaggaccaactcctca
Pig_ZFP36L1_qPCR_F_109	acggcaccggcaccttccct
Pig_ZFP36L1_qPCR_R_109	tggcgacacctctcccaaagggg

**Table 2:** List of genes upregulated by GABA related to positive regulation of erythrocyte differentiation and synergistically upregulated by GABA and PIP related to immune system process

Items	Gene	GABA	Piperine	G+P
Positive regulation of erythrocyte differentiation	*ZFP36L1*	1.66	1.01	1.20
	*STAT1*	1.54	1.04	1.02
	*HIF1A*	1.62	1.03	1.08
	*STAT1*	1.54	1.04	1.02
	*HMGB2*	1.56	−1.04	1.41
	*LDB1*	1.90	1.17	1.19
	*INHBA*	1.78	1.12	−1.42
Immune system process	*DEFB1*	1.26	1.14	1.51
	*TRIM8*	−1.03	1.44	2.31
	*ADAM15*	2.05	1.05	2.17
	*TEC*	1.16	1.19	1.53
	*CAV1*	1.35	1.17	1.52
	*PLCL2*	1.12	−1.05	1.53
	*CD3E*	1.96	−1.04	2.07
	*HMGB2*	−1.04	−1.04	1.61
	*GATA3*	−1.39	−1.25	1.63
	*CCNB2*	1.09	−1.10	1.50
	*ARID4A*	1.02	1.04	1.59
	*C4A*	1.16	1.27	1.59
	*TMEM173*	1.56	1.41	1.94
	*BPIFA1*	1.38	−1.11	2.47
	*TMEM173*	1.56	1.41	1.94
	*TREM2*	1.25	1.03	1.53
	*ENPP2*	−1.28	1.14	1.50
	*ITM2A*	−1.05	1.10	1.86
	*RAB29*	1.12	1.02	1.78
	*CD3E*	1.96	−1.04	2.07
	*OAS2*	1.02	1.06	1.66
	*IRAK1*	1.19	−1.10	1.51
	*THRA*	1.29	1.14	1.52
	*HMGB2*	−1.04	−1.04	1.61
	*TOP2A*	1.22	−1.05	1.59
	*SLA-DOA*	1.43	1.03	1.51
	*KMT2E*	1.14	−1.06	1.71
	*REST*	−1.03	−1.00	1.61
	*BPIFA1*	1.38	−1.11	2.47
	*GCNT1*	−1.08	1.26	1.56
	*CCL14*	−1.59	−1.56	2.70

Genes’ fold changes (FCs) compared to control are listed for GABA, PIP, and GABA/PIP (G+P) co-treatment.

GABA, gamma-aminobutyric acid; PIP, piperine; *ZFP36L1*, SPF36 ring finger protein like 1; *STAT1*, signal transducer and activator of transcription 1; *HIF1A*, hypoxia-inducible factor 1-alpha; *HMGB2*, high mobility group protein B2; *LDB1*, LIM domain-binding protein 1; *INHBA*, inhibin subunit beta A; *DEFB1*, defensin beta 1; *TRIM8*, tripartite motif containing 8; *ADAM15*, ADAM metallopeptidase domain 15; *TEC*, Tec protein ty-rosine kinase; *CAV1*, caveolin 1; *PLCL2*, phospholipase C like 2; *CD3E*, CD3e molecule; *GATA3*, GATA binding protein 3; *CCNB2*, cyclin B2; *ARID4A*, AT-rich interaction domain 4A; *C4A*, complement C4A (Rodgers Blood Group); *TMEM173*, transmembrane protein 173; *BPIFA1*, BPI fold containing family A member 1; *TREM2*, triggering receptor expressed on myeloid cells 2; *ENPP2*, ectonucleotide pyrophosphatase/phosphodiesterase 2; *ITM2A*, integral membrane protein 2A; *RAB29*, ras-related protein Rab-7L; *OAS2*, 2′-5′-oligoadenylate synthetase 2; *IRAK1*, interleukin-1 receptor-associated kinase 1; *THRA*, thyroid hormone receptor alpha; *TOP2A*, DNA topoisomerase II alpha; *SLA-DOA*, major histocompatibility complex, classII Do alpha; *KMT2E*, myeloid/lymphoid or mixed-lineage leukemia 5; *REST*, RE1-silencing transcription factor; *GCNT1*, glucosami-nyl (N-acetyl) transferase 1; *CCL14*, C-C motif chemokine ligand 14.
